# MRI T2w Radiomics-Based Machine Learning Models in Imaging Simulated Biopsy Add Diagnostic Value to PI-RADS in Predicting Prostate Cancer: A Retrospective Diagnostic Study

**DOI:** 10.3390/cancers16172944

**Published:** 2024-08-23

**Authors:** Jia-Cheng Liu, Xiao-Hao Ruan, Tsun-Tsun Chun, Chi Yao, Da Huang, Hoi-Lung Wong, Chun-Ting Lai, Chiu-Fung Tsang, Sze-Ho Ho, Tsui-Lin Ng, Dan-Feng Xu, Rong Na

**Affiliations:** 1 Department of Urology, Ruijin Hospital, Shanghai Jiao Tong University School of Medicine, Shanghai 200025, China; 2 Department of Surgery, School of Clinical Medicine, LKS Faculty of Medicine, The University of Hong Kong, Hong Kong, China; 3 Department of Surgery, Queen Mary Hospital, Hong Kong, Chinalct729@ha.org.hk (C.-T.L.);

**Keywords:** prostate cancer, multi-parametric magnetic resonance imaging, prostate imaging reporting and data system, radiomics, machine learning, biopsy

## Abstract

**Simple Summary:**

Prostate mpMRI is currently the most widely used image diagnosis approach to detect prostate cancer, while the PI-RADS system was developed to standardize and improve the accuracy of suspicious lesion identification on MRI. However, there still remain several limitations including inter-individual inconsistencies and naked-eye insufficiency. This study aims to apply AI technology to image interpretation to enhance diagnostic efficiency and explore the use of T2-weighted image-based stimulated biopsy in predicting prostate cancer (PCa). Using 820 lesions from The Cancer Imaging Archive database and 83 lesions from Hong Kong Queen Mary Hospital, we constructed 18 machine-learning models based on three algorithms and conducted both internal and external validation. We found that the logistic regression-based model provides additional diagnostic value to the PI-RADS in predicting PCa.

**Abstract:**

Background: Currently, prostate cancer (PCa) prebiopsy medical image diagnosis mainly relies on mpMRI and PI-RADS scores. However, PI-RADS has its limitations, such as inter- and intra-radiologist variability and the potential for imperceptible features. The primary objective of this study is to evaluate the effectiveness of a machine learning model based on radiomics analysis of MRI T2-weighted (T2w) images for predicting PCa in prebiopsy cases. Method: A retrospective analysis was conducted using 820 lesions (363 cases, 457 controls) from The Cancer Imaging Archive (TCIA) Database for model development and validation. An additional 83 lesions (30 cases, 53 controls) from Hong Kong Queen Mary Hospital were used for independent external validation. The MRI T2w images were preprocessed, and radiomic features were extracted. Feature selection was performed using Cross Validation Least Angle Regression (CV-LARS). Using three different machine learning algorithms, a total of 18 prediction models and 3 shape control models were developed. The performance of the models, including the area under the curve (AUC) and diagnostic values such as sensitivity, specificity, positive predictive value (PPV), and negative predictive value (NPV), were compared to the PI-RADS scoring system for both internal and external validation. Results: All the models showed significant differences compared to the shape control model (all *p* < 0.001, except SVM model PI-RADS+2 Features *p* = 0.004, SVM model PI-RADS+3 Features *p* = 0.002). In internal validation, the best model, based on the LR algorithm, incorporated 3 radiomic features (AUC = 0.838, sensitivity = 76.85%, specificity = 77.36%). In external validation, the LR (3 features) model outperformed PI-RADS in predictive value with AUC 0.870 vs. 0.658, sensitivity 56.67% vs. 46.67%, specificity 92.45% vs. 84.91%, PPV 80.95% vs. 63.64%, and NPV 79.03% vs. 73.77%. Conclusions: The machine learning model based on radiomics analysis of MRI T2w images, along with simulated biopsy, provides additional diagnostic value to the PI-RADS scoring system in predicting PCa.

## 1. Introduction

Prostate cancer (PCa) is one of the most prevalent cancers among males worldwide, with the highest incidence and leading to the second highest number of deaths [1,2]. In recent decades, the prevalence of prostate cancer has been on the rise in China. One notable difference is that the ratio of mortality-to-morbidity in prostate cancer is higher in China compared with Western countries [3,4].

Current diagnostic methods for detecting PCa typically involve various tests, such as prostate-specific antigen (PSA), prostate health index (*phi*), digital rectal examination (DRE), and multiparametric magnetic resonance imaging (mpMRI) [5,6,7]. These tests are used to identify potential cases of PCa. If the results indicate suspicion of PCa, a subsequent invasive biopsy, considered the gold standard in diagnostics, is performed for confirmation.

However, as an invasive procedure, prostate biopsy carries the risk of various side effects, such as bleeding, pain, infection, and, in severe cases, life-threatening sepsis [8]. Therefore, it is essential to assess the necessity of subjecting a patient to invasive biopsy. For individuals requiring biopsy, efforts should be made to enhance the accuracy of the procedure to mitigate the risk of false negatives and subsequent unnecessary interventions such as active surveillance or repeat biopsies.

The currently most widely used imaging method, mpMRI, provides a comprehensive depiction of different physiological and anatomical characteristics through the utilization of various imaging sequences. MRI-Targeted biopsy is one of the approaches to reduce the misclassification of clinically significant prostate cancer in men with MRI-visible lesions. This method involves superimposing T2w MRI images onto real-time ultrasound scans of the prostate, enabling clinicians to identify and biopsy suspicious. However, due to limitations inherent in mpMRI scans, current T2w targeted biopsy did not show a significantly improved PCa detection rate compared to systematic biopsy (51.5% vs. 52.5%, respectively) [9,10].

To standardize and improve the accuracy of suspicious lesion identification on MRI, the Prostate Imaging Reporting and Data System version 2.1 (PI-RADS v2.1) was developed. This system has demonstrated promising value in clinical practice [11]. Nevertheless, there still remain several limitations. Firstly, the PI-RADS assessment highly depends upon the individual interpreting the images, resulting in potential inconsistencies. Specifically, experienced radiologists tend to exhibit superior performance compared to their less experienced counterparts, and a group of radiologists collectively performs better than a single radiologist [12]. Secondly, there are certain features that are not visible to the naked eye and therefore cannot be directly observed or assessed [13]. These limitations can potentially compromise the accuracy and reliability of prostate cancer diagnoses.

Recently, the wide application of machine learning (ML) and artificial intelligence (AI) approaches has made it possible to overcome these disadvantages by establishing diagnostic tools based on radiomics data. Radiomics focuses on the extraction and analysis of a vast array of quantitative imaging features from medical images, enabling the analysis of characteristics that may be imperceptible to the naked eye and fostering a more objective approach. ML methods, which are increasingly being incorporated into radiomic studies, are used to analyze these high-dimensional features. Specifically, ML is a field of AI that focuses on the development of algorithms and models that enable computers to learn and make predictions or decisions without being explicitly programmed for each specific task. This combination of radiomics and ML has proved valuable for constructing medical prediction models [14,15,16].

Previous studies have already examined the diagnostic potential of radiomics in PCa diagnosis prior to biopsy by segmenting suspected areas [16,17,18,19]. In this study, our primary objective is to assess the efficacy of MRI T2w-based simulated biopsy. We aim to accomplish this by constructing a biopsy trajectory radiomics-based machine learning model for PCa prediction and comparing its incremental value to the current PI-RADS scoring system.

## 2. Method

**Figure 1:**
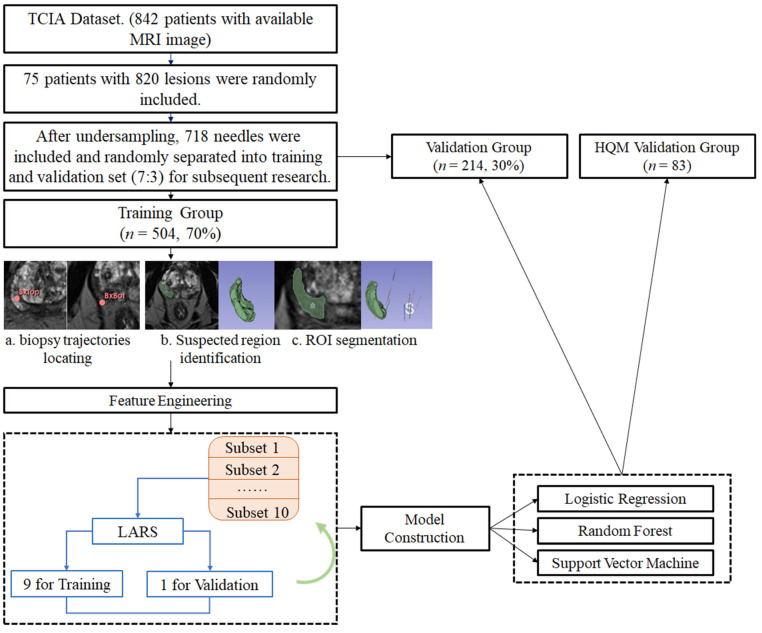
Workflow of models’ construction and validation. Figure 1 describes the workflow of this study: (1) The MRI images were obtained from two sources: The Cancer Imaging Archive (TCIA) Database Collection (75 patients, 820 lesions) and Hong Kong Queen Mary Hospital (8 patients, 83 lesions); (2) The 820 lesions from TCIA were undersampled to 718 lesions and randomly separated to training group (*n* = 504, 70%) and validation group (*n* = 214, 30%); (3) Initial regions of interest (ROIs) were manually delineated on the MRI image, as indicated by the red markers (BxTop and BxBot); (4) ROIs were refined and segmented. The green overlay highlights the segmented prostate region; (5) A set of radiomic features was screened using the LARS (Least Angle Regression) algorithm. This step identified the most relevant features for prostate cancer prediction; (6) The extracted features were used to train logistic regression, random forest, and support vector machine models; (7) Performance of models was both validated internally and externally.

This retrospective research involved lesion-to-lesion analysis. All the biopsy trajectories from two sources were delineated, and radiomic features were extracted from the MRI T2w images (T2W). After the feature selection process, ML algorithms were used to construct and validate prediction models. Figure 1 shows the workflow of this research. 

### 2.1. Patients and Data Collection

Data were retrospectively obtained from two sources: TCIA (The Cancer Imaging Archive) Database PROSTATE-US-MR Collection and Hong Kong Queen Mary Hospital. The PROSTATE-US-MR collection included a total of 1151 patients, of which 842 patients underwent MRI scans. Clinical characteristics including PSA, PI-RADS, and T2W MRI images were recorded for these 842 patients. Biopsy tracks were also labeled. MR imaging was performed on a 3 Tesla Trio, Verio, or Skyra scanner (Siemens, Erlangen, Germany). From the 842-patient collection, 75 patients with 820 available needle tracks were randomly selected for further model construction and internal validation. Additionally, 83 needles from 8 patients who experienced MRI-targeted biopsy at Queen Mary Hospital from June 2022 to October 2023, with available clinical variables and track recordings, were included for external validation. The study was approved by the Institutional Review Board of the University of Hong Kong/Hospital Authority Hong Kong West Cluster (UW20-462). All participants provided written informed consent to take part in the study.

### 2.2. PI-RADS

The PI-RADS score was evaluated at the needle level. When the biopsy trajectory intersected the suspected region, the score was determined based on the highest PI-RADS score assigned to any part of that region. The final PI-RADS score of suspected regions was a comprehensive evaluation considering the dataset results, as well as assessments by our own radiologists and urologists. A PI-RADS score ≥ 4 was considered a positive prediction, while ≤3 is regarded as negative. 

### 2.3. Segmentation and Feature Extraction

#### 2.3.1. ROI Segmentation

Biopsy trajectories were generated using the 3D Slicer software, employing the “draw tube” tool with a radius setting of 1.00. Along with each recorded trajectory, the suspected region (identified as suspicious in the dataset or by our researchers, consistent with the region of PI-RADS score region) was delineated. 

#### 2.3.2. Pyradiomics

The “PyRadiomics” package, developed by Harvard Medical School’s Computational Imaging and Bioinformatics Lab, is widely adopted in contemporary radiomics research. It offers a wide range of radiomics features that are compliant with the Image Biomarker Standardization Initiative (IBSI), ensuring consistency and reproducibility. The features provided include First-order Statistics, Shape-based Features, Texture Features, and Wavelet Filtered Features. First-order Statistics describe the distribution of voxel intensities within the image region, without considering spatial relationships; Shape-based Features quantify the geometric properties of the segmented region; Texture Features include Gray Level Co-occurrence Matrix (GLCM), Gray Level Run Length Matrix (GLRLM), Gray Level Size Zone Matrix (GLSZM), Gray Level Dependence Matrix (GLDM) and Neighboring Gray Tone Difference Matrix (NGTDM). These texture features capture various aspects of the image’s texture by analyzing the relationships and patterns of pixel intensities. Wavelet Filtered Features describe the characteristics of the region of interest by applying wavelet transformations, which can capture details at multiple scales. However, due to the uninterpretability of Wavelet Filtered Features, they were not included in the model construction. 

There was also a previous study that constructed “MaZda” package for radiomic features extraction [20]. However, it is an older software package that predates the formalization of the IBSI standards. While MaZda includes a comprehensive set of texture features, it was not explicitly designed with IBSI standards in mind. Furthermore, MaZda is currently less widely used than PyRadiomics, with limited updates and community support, making it less suitable for ensuring reproducibility and consistency in modern research.

#### 2.3.3. Feature Extraction

To enhance the robustness of the extracted features, several steps were performed, including the following image preprocessing and feature extraction were conducted using 3D Slicer (version 5.2.1): (1) The images were standardized by using “histogram matching” to eliminate the intensity variation; (2) we applied “N4ITK MRI Bias correction” to adjust the potentially corrupted MRI images caused by bias field signal; (3) the images were resampled to a voxel size of 1 × 1 × 1 mm to standardize voxel spacing and voxel intensity values were discretized using a fixed bin width of 25 HU; (4) based on the Image Biomarker Standardization Initiative (IBSI), 107 features were extracted by “Pyradiomics” package; (5) The images of 20 patients from TCIA dataset with 333 needles were re-evaluated by both two doctors twice, both intra- and inter-doctor intraclass correlation coefficient (ICC) for each feature were calculated and the variables with an ICC > 0.75 were included. Finally, 103 features were included for further model construction.

### 2.4. Model Construction

#### 2.4.1. Data Undersampling

Commonly, only a portion of the biopsy needles from a single PCa patient will yield positive results. The imbalance in prostate cancer findings is more pronounced at the patient level compared to the needle level. Thus, in this research, we mainly focus on the needle level. The imbalance in the numbers of positive needles (*n* = 363) and negative needles (*n* = 457) could potentially impact the model’s performance (Table 1). To address this issue, we employed the “ROSE” package in R for undersampling the data. This approach aims to create a more balanced dataset for data screening and model training, thereby preventing the predictive ability of the model from being influenced by dataset imbalance and potentially causing bias. We randomly selected a portion of the negative needles to make the number close to that of the positive group for further model construction.

#### 2.4.2. Feature Selection

**Figure 2:**
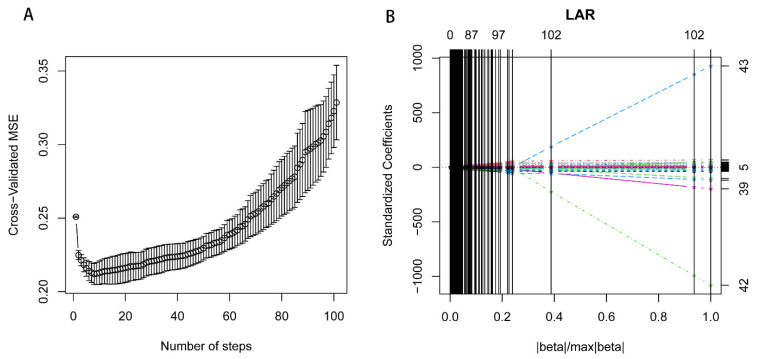
Ten-fold cross-validation least angle regression for feature selection. Figure 2 Plots describing the 10-fold cross-validation least angle regression (cv-LARS) based feature selection process and exhibits the results. (**A**). illustrating the changes in cross-validated Mean Squared Error (MSE) with the number of steps. At the 8th step, the LARS algorithm reaches the minimum MSE. (**B**). exhibiting the solution path plot of 10-fold cv-LARS.

Feature selection was performed using the step-forward 10-fold Cross Validation Least Angle Regression (CV-LARS) algorithm on the training set. Model performance was evaluated using the mean standard error (MSE). At the 8th step of the algorithm, the LARS achieved the lowest MSE, resulting in the extraction of 7 relevant features (Figure 2). Notably, the earlier a feature is included in the algorithm, the more significant it is in predicting the outcome.

#### 2.4.3. Predictive Models and Control Models

To construct predictive models, we included the selected features along with a PI-RADS score. Three distinct machine learning classifiers, namely logistic regression (LR), random forest (RF), and support vector machine (SVM), were trained to predict the presence of PCa. Starting with the first feature among the seven, we gradually added more features to create multiple models. We compared the performance of these models to determine the optimal one. 

In addition, we developed two control models for comparative analysis: a shape control model and a PI-RADS model. The “shape control model” was constructed by setting all non-shape-related features to zero, focusing solely on the impact of ROI shape in predicting PCa. This helped us eliminate the potential biases introduced by manual ROI drawing. The “PI-RADS model” was created using only the PI-RADS score as input variables. This model allowed us to assess the incremental value of radiomic features in predicting PCa beyond the information provided by current criteria, PI-RADS, alone.

### 2.5. Statistical Analysis

The variables in the training dataset and validation dataset were compared using the Wilcoxon signed-rank test. The performance of three machine learning models was evaluated by sensitivity, specificity, positive predictive value (PPV), and negative predictive value (NPV). The comparison between the different models (LR, RF, and SVM) was evaluated using Receiver Operating Characteristic (ROC) curves. The Area Under the Curve (AUC) was compared using DeLong’s test. A two-tail *p*-value < 0.05 was considered significant. All the statistical analysis and model construction were performed using R version 4.2.2 [21].

## 3. Results

### 3.1. Patients

A total of 75 patients with 820 biopsy needles were finally included in this study, as indicated in Table 1. Among these, 363 needles (44.27%) were pathologically confirmed to be malignant, while 457 needles (55.73%) were pathologically proven to be benign. Following the undersampling process, the distribution of the target variable was balanced, with 363 needles (50.56%) classified as malignant and 355 needles (49.44%) classified as benign. 

Subsequently, the resampled data were randomly divided into a training group and a validation group, with a ratio of 7:3, as shown in Table 1. The training group consisted of 504 needles, while the validation group consisted of 214 needles. There were no significant differences observed between these two groups in terms of PSA, biopsy outcome, GG, PI-RADS, or MRI results (*p* = 0.65, 0.98, 0.88, 0.2, and 0.42, respectively, Table 1 and Supplementary Table S1).

### 3.2. Feature Screening

For feature selection, we utilized the step-forward CV-LARS algorithm on the training set. Ultimately, at the 8th step, LARS achieved the lowest MSE (Supplementary Table S2). As a result, seven relevant features were extracted. The seven relevant features that were extracted are as follows, sorted in the order of inclusion according to LARS: (1) “originalglcmSumEntropy”; (2) “originalgldmDependenceNonUniformity”; (3) “originalfirstorderMaximum”; (4) “originalglrlmRunLengthNonUniformity”; (5) “originalglszmSizeZoneNonUniformity”; (6) “originalshapeSurfaceArea”; (7) “originalshapeMaximum2DDiameterColumn”.

### 3.3. Model Performance

#### TCIA Dataset

Model construction was performed at each step of the LARS algorithm, and the performance was internally validated according to the TCIA Database as presented in Table 2 and Table 3.

When comparing with the shape control model, all the models demonstrated significant differences (all *p* < 0.001, except SVM model PI-RADS+2 Features *p* = 0.004, SVM model PI-RADS+3 Features *p* = 0.002). Furthermore, both the six LR prediction models and six SVM prediction models showed significant superiority when compared to PI-RADS alone (*p* < 0.001). Additionally, five out of the six RF prediction models performed better than PI-RADS alone (*p* < 0.05), except for the RF model PI-RADS+3 Features (*p* = 0.220).

When comparing the different models constructed by the same algorithm and different feature numbers, the model based on LR incorporated 3 radiomic features and yielded AUC = 0.838, sensitivity = 76.85%, and specificity = 77.36%. There are no significant differences in performance among LR models with 3, 4, 5, 6, or 7 features. Since models with fewer features are generally less prone to overfitting, which occurs when a model learns noise and details from the training data that do not generalize well to new data, we selected the LR model with 3 radiomic features as the best LR model for further research due to its simplicity and reduced risk of overfitting. Similarly, the best RF model exploited 2 radiomic features, yielding AUC = 0.775, and the best SVM model exploited 6 radiomic features with an AUC = 0.827 (Table 3).

**Figure 3:**
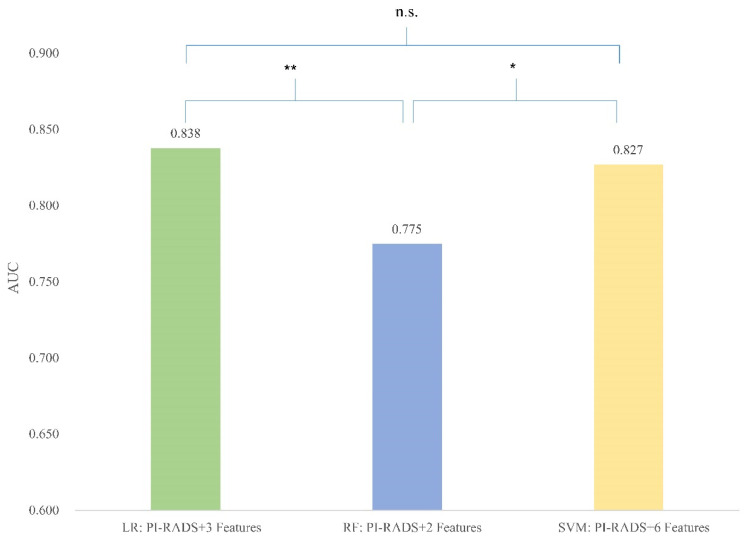
Comparison between best-performing models. Figure 3 column plots showing the area under the curve (AUC) of three best-performing models based on different algorithms. * *p* < 0.05; ** *p* < 0.01; n.s. indicates non-significance.

In the further comparison, both the LR (3 features) model and the SVM (6 features) model showed significantly better predictive value than the RF (2 features) model (Figure 3, compared to the LR model *p* = 0.003, compared to the SVM model *p* = 0.01).

### 3.4. HQM Validation Dataset

**Figure 4:**
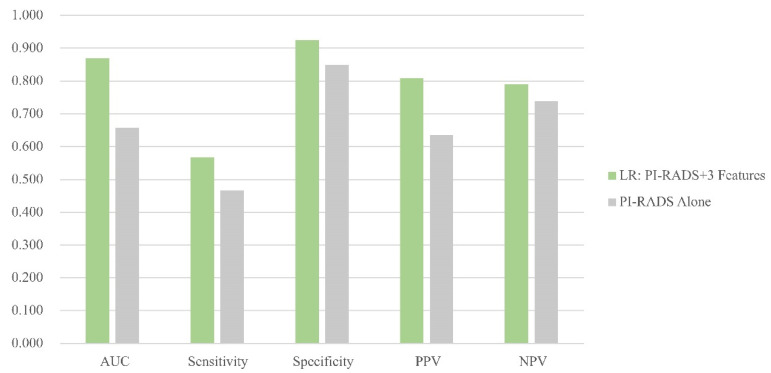
Comparison between the optimal model and PI-RADS in HQM set. Figure 4 column plot showing the area under the curve of the logistic regression model (3 features + PI-RADS).

External validation was performed in the HQM dataset based on eighty-three needles from 8 patients who experienced MRI-targeted prostate biopsy at Hongkong Queen Mary Hospital. The LR (3 features) model was compared to PI-RADS. The radiomics model outperforms PI-RADS in predictive value with AUC 0.870 vs. 0.658, sensitivity 56.67% vs. 46.67%, specificity 92.45% vs. 84.91%, PPV (positive predictive value) 80.95% vs. 63.64% and NPV (negative predictive value) 79.03% vs. 73.77%. Figure 4 is a visualization of the comparison between the LR (3 features) model and PI-RADS for the prediction of PCa.

## 4. Discussion

This study explored the prospective utility of simulated prostate biopsy using MRI T2-weighted (T2w) images to predict PCa before an actual invasive biopsy. We developed a diagnostic radiomics model based on selected MRI T2w features combined with PI-RADS, demonstrating superior performance compared to PI-RADS alone in predicting PCa.

PI-RADS stands out as the predominant system for standardizing the assessment of mpMRI in the evaluation of potential prostate disease. It classifies lesions into five levels based on their characteristics, offering a relatively standardized protocol for radiologists to interpret prostate MRI images. However, PI-RADS still has some limitations, such as variability in the interpretation of inter- or intra-radiologists with different levels of experience, and the potential features to be imperceptible to the naked eye, leading to unavoidable false-negative predictions. For instance, Moon Hyung Choi et al. reported variations in inter-reader agreements of PI-RADS scores among radiologists, with accuracy ranging from 54.5% to 82.6% and inter-reader agreements ranging from poor to good [12,13]. Moldovan, PC et al. conducted a meta-analysis based on 48 studies (involving 9613 patients) and found that the inter-observer reproducibility of existing scoring systems still requires improvement [22]. Samuel Borofsky et al. discovered that 16% of malignant lesions were missed on mpMRI, with 58% of these lesions either not visible or characterized as benign [23]. Additionally, in terms of MRI-targeted biopsy, Williams et al. noted that most misses are attributed to errors in lesion targeting, underscoring the importance of accurate co-registration and targeting techniques [24].

Radiomics, as a novel methodology, focuses on the quantitative and objective evaluation of medical images. It assists radiologists in achieving precise diagnoses of prostate cancer, thereby reducing the need for unnecessary follow-up procedures [25].

The radiomics research pipeline basically comprises several integral procedures, image preprocessing, feature extraction and selection, model construction, and validation. To enhance image homogeneity, we executed histogram calibration and N4ITK MRI bias correction. Subsequently, we extracted 107 features using the “Pyradiomics” package, adhering to the Image Biomarker Standardization Initiative (IBSI) to enhance robustness. During the feature selection process, we applied 10-fold cross-validation LARS rather than LASSO (least absolute shrinkage and selection operator), which is more commonly used in studies with high-dimensional features [26]. LARS is an iterative algorithm that adds features into the model one at a time based on their correlation with the residuals of the response variable. At each step, LARS adds the feature most correlated with the residuals of the model. The significance of a feature is indicated by how early it is included in the algorithm, as highly correlated features with the outcome are added first. This automated and objective process ensures that the most predictive features are prioritized, so as to mitigate the potential risk of overfitting by gradually incorporating fewer features step by step to meet optimal performance.

Previous studies have proved traditional machine learning algorithms’ capability in constructing radiomics models focusing on prostatic areas. Hou et al. developed several radiomics models based on three algorithms, LR, RF as well as SVM to predict lymph node invasive. These models outperformed the MSKCC nomogram and helped to spare a large number of unnecessary extended pelvic lymph node dissections [27]. Zheng et al. trained an SVM model combining manually crafted radiomics features and clinical characteristics to predict biopsy results for patients with negative MRI findings. However, this research only included 330 lesions, with no external validation [28]. Although there have already been encouraging results proving the additional value of radiomics model in prostate cancer diagnosis, to our knowledge, only several studies validated their models in external cohorts [25].

In this study, we utilized the TCIA Database PROSTATE-US-MR Collection, which contains a large number of available recorded biopsy trajectories, corresponding PI-RADS scores, and pathological outcomes. This database enabled us to conduct the lesion-to-lesion study at the needle level, facilitating the training and validation of our models with a large sample size. Based on the 820 lesions from 75 patients randomly selected, we constructed 18 different prediction models by harnessing 3 machine learning algorithms. Remarkably, the LR radiomics model demonstrated optimal performance with the inclusion of only three features. When integrated with PI-RADS, it surpassed the performance of PI-RADS alone in PCa detection, validated both internally within the TCIA database and externally in the HQM cohort.

This outcome aligns with prior research, affirming the additional value of the radiomics model to clinical parameters, particularly PI-RADS. There was a previous study analyzing the correlation between PI-RADS and the radiomic features extracted from prostate MRI images using the qMaZda software (https://qmazda.p.lodz.pl/pms/SoftwareQmazda.html, accessed on 25 July 2024) [29]. Gibała et al. demonstrated that SVM models trained on texture features extracted from mpMRI images can achieve accurate diagnostic performance in detecting PCa. However, it is important to note that their study utilized qMaZda, an older software package that predates the formalization of the IBSI standards. Additionally, due to the limited sample size (*n* = 92), they relied on internal cross-validation without performing external validation. Building on this prior work, we refined our research methodology by splitting the patient-level images into needle-level datasets. This approach allowed us to significantly increase the sample size, thereby enhancing the robustness and generalizability of our results. Moreover, it underscores the capabilities of radiomics even in scenarios of incomplete lesion segmentation. Unlike many previous radiomics studies that manually delineate the complete suspicious region [8,18,25,30,31,32,33], our approach demonstrates the potential of radiomics within the biopsy trajectories, which are incomplete segmented lesions. Leveraging the “shape control model”, we effectively mitigated potential shape bias introduced by manual crafting. This was evident in the significant difference observed between the LR model and the shape control model. The only three radiomic features incorporated into the LR model, namely “original glcm Sum Entropy”, “original gldm Dependence NonUniformity”, and “original first-order Maximum”, illustrate the texture characteristics of the lesion. Our study indicates a promising future for simulated biopsy in prostate medical imaging, potentially enhancing the utility of the current PI-RADS system. Clinicians may consider conducting a simulated biopsy on MRI images for patients with suspicious PCa, aiding in identifying individuals who genuinely require invasive biopsy procedures. Furthermore, integrating the modeled suspected lesion into MRI-targeted biopsy procedures could enhance diagnostic accuracy. Further external validation in a much larger dataset is essential to evaluate the diagnostic performance and robustness of our model. Additionally, conducting large randomized controlled trials (RCTs) will be necessary to establish the efficacy and reliability of this approach in diverse clinical settings. If validated, this model could be developed into a software program accessible on both PC and mobile devices. Based on the widespread use of online medical imaging tools, such a program could be seamlessly integrated into current clinical workflows. In the future, clinicians, and potentially patients themselves, could simulate biopsy tracks on their devices, reducing the public health burden by minimizing unnecessary invasive procedures.

This study has several limitations. Firstly, only T2w images were incorporated due to the sole availability of this sequence in the PROSTATE-US-MR collection; DWI and ADC sequences were not accessible. Nonetheless, a prior radiomics study concentrating on predicting Gleason grade groups exclusively based on the T2w sequence yielded promising results [34]. Additionally, our model demonstrated robust performance in the external validation set. Nevertheless, for a more comprehensive understanding, further research encompassing multiple sequences is warranted. Secondly, to bolster the robustness and reliability of this model, it is advisable to conduct future multicenter validations with an expanded participant pool and a prospective design. In this study, the dataset was split into training and validation sets comprising 718 images, meaning that some validation lesions could have originated from the same patients present in the training set, potentially introducing bias. To mitigate this issue, we implemented several measures to ensure the independence of each needle track: (1) Biopsy trajectories on MRI images were generated by experienced urologists who were blinded to the pathology outcomes, ensuring unbiased assessments. (2) Rigorous standardization procedures, including histogram calibration and N4ITK MRI bias correction, were applied to all images to minimize variability and enhance data consistency. (3) During model construction, only PI-RADS scores of needle tracks and radiomic features were used, with deliberate exclusion of overall patient characteristics such as PSA or prostate volume. Additionally, we conducted an analysis with the training dataset split by subjects, as detailed in Supplementary Table S5. Following the application of the CV-LARS feature selection process, 10 features were chosen for further model development (Supplementary Table S6, Supplementary Figure S1). The resulting logistic regression (LR) model, which combined PI-RADS with 7 selected features, achieved an AUC of 0.864 (95% CI: 0.807–0.921), with a sensitivity of 81.25% and a specificity of 82.05% (Supplementary Table S7). These metrics surpassed those of the needle-level model. Furthermore, the optimal LR model demonstrated significantly superior performance compared to PI-RADS alone in external validation (*p* < 0.001, Supplementary Figure S2). While the features selected in the subject-based model differed from those in the needle-level model, the overall performance metrics remained consistent, thereby reinforcing the robustness of our original needle-level approach. This validation suggests that our approach is appropriate for the research objectives and does not suffer from significant data leakage or bias. However, for broader clinical applications, it will be crucial to further enhance the model’s robustness and generalizability through larger, subject-based independent validation datasets. Lastly, this study exclusively considered PCa as the response variable, and clinical significance (csPCa) was not taken into account. However, previous research on the treatment patterns for PCa in China indicates that only a mere 2.33% of low-risk PCa patients opted for active surveillance or observation, despite AS (active surveillance) being widely recommended for such cases in guidelines [35]. This phenomenon may be attributed to cultural factors making it challenging to accept a malignancy without intervention and the relatively limited access to persistent healthcare and follow-up services.

## 5. Conclusions

Based on internal and external validation, we demonstrated that the logistic regression model incorporating three MRI T2w radiomic features and PI-RADS performs well in diagnosing prostate cancer. The machine learning and MRI T2w-based simulated biopsy radiomics model add diagnostic value to PI-RADS in predicting prostate cancer. In the prospective future, clinicians may contemplate performing a simulated biopsy on MRI images for patients with suspected prostate cancer, assisting in identifying those who truly necessitate invasive biopsy procedures.

## Figures and Tables

**Table 1:** Patients’ Characteristics.

	Patient Level Charcteristics	Needle Level before Resample	Needle Level after Resample	Needle Level Training	Needle Level Validation	Needle Level *p* Value *
Number	75	820	718	504	214	/
PSA	6.50 (4.50~9.50)	6.90 (5.40~11.00)	6.50 (5.40~12.00)	6.90 (5.30~11.00)	6.50 (5.40~12.00)	0.65
Prostate Cancer						0.98
Yes	64 (85.33%)	363 (44.27%)	363 (50.56%)	255 (50.60%)	108 (50.47%)	/
No	11 (14.67%)	457 (55.73%)	355 (49.44%)	249 (49.40%)	106 (49.53%)	/
Gleason Grade Group						0.88
Not PCa	11 (14.67%)	457 (55.73%)	355 (49.44%)	249 (49.40%)	106 (49.53%)	/
GG1	16 (21.33%)	184 (22.44%)	184 (25.63%)	129 (25.6%)	55 (25.70%)	/
GG2	22 (29.33%)	98 (11.95%)	98 (13.65%)	67 (13.29%)	31 (14.49%)	/
GG3	14 (18.67%)	35 (4.27%)	35 (4.87%)	24 (4.76%)	11 (5.14%)	/
GG4	3 (4.00%)	23 (2.80%)	23 (3.20%)	17 (3.37%)	6 (2.80%)	/
GG5	9 (12.00%)	23 (2.80%)	23 (3.20%)	18 (3.57%)	5 (2.34%)	/

PSA: prostate-specific antigen; PCa: prostate cancer; GG: grade group. * Wilcoxon signed-rank test comparing training dataset and validation dataset.

**Table 2:** Performance of different models.

	Variables	AUC	Sensitivity	Specificity	PPV	NPV	*p* Value (vs. PI-RADS Control) *	*p* Value (vs. Shape Control) *
LR	Shape Control	0.659 (0.596–0.723)	61.11%	70.75%	68.04%	64.10%	/	/
	PI-RADS Alone	0.701 (0.640–0.763)	67.59%	72.64%	71.57%	68.75%	/	/
	PI-RADS+2 Features	0.835 (0.779–0.89)	74.07%	77.36%	76.92%	74.55%	<0.001	<0.001
	PI-RADS+3 Features	0.838 (0.783–0.894)	76.85%	77.36%	77.57%	76.64%	<0.001	<0.001
	PI-RADS+4 Features	0.835 (0.780–0.891)	76.85%	76.42%	76.85%	76.42%	<0.001	<0.001
	PI-RADS+5 Features	0.833 (0.777–0.889)	79.63%	78.30%	78.90%	79.05%	<0.001	<0.001
	PI-RADS+6 Features	0.840 (0.784–0.896)	81.48%	78.30%	79.28%	80.58%	<0.001	<0.001
	PI-RADS+7 Features	0.841 (0.785–0.896)	81.48%	79.25%	80.00%	80.77%	<0.001	<0.001
RF	Shape Control	0.617 (0.552–0.682)	58.33%	65.09%	63.00%	60.53%	/	/
	PI-RADS Alone	0.701 (0.640–0.763)	67.59%	72.64%	71.57%	68.75%	/	/
	PI-RADS+2 Features	0.776 (0.720–0.831)	84.26%	70.75%	74.59%	81.52%	0.005	<0.001
	PI-RADS+3 Features	0.743 (0.684–0.801)	77.78%	70.75%	73.04%	75.76%	0.220	<0.001
	PI-RADS+4 Features	0.766 (0.709–0.822)	82.41%	70.75%	74.17%	79.79%	0.042	<0.001
	PI-RADS+5 Features	0.766 (0.709–0.823)	80.56%	72.64%	75.00%	78.57%	0.042	<0.001
	PI-RADS+6 Features	0.790 (0.735–0.844)	81.48%	76.42%	77.88%	80.20%	0.008	<0.001
	PI-RADS+7 Features	0.794 (0.740–0.849)	81.48%	77.36%	78.57%	80.39%	0.005	<0.001
SVM	Shape Control	0.655 (0.593–0.728)	55.56%	75.47%	69.77%	62.50%	/	/
	PI-RADS Alone	0.701 (0.640–0.763)	67.59%	72.64%	71.57%	68.75%	/	/
	PI-RADS+2 Features	0.771 (0.714–0.827)	81.48%	72.64%	75.21%	79.38%	<0.001	0.004
	PI-RADS+3 Features	0.780 (0.725–0.835)	84.26%	71.70%	75.21%	81.72%	<0.001	0.002
	PI-RADS+4 Features	0.799 (0.745–0.853)	83.33%	76.42%	78.26%	81.82%	<0.001	<0.001
	PI-RADS+5 Features	0.794 (0.74–0.848)	82.41%	76.42%	78.07%	81.00%	<0.001	<0.001
	PI-RADS+6 Features	0.827 (0.776–0.878)	85.19%	80.19%	81.42%	84.16%	<0.001	<0.001
	PI-RADS+7 Features	0.818 (0.766–0.870)	83.33%	80.19%	81.08%	82.52%	<0.001	<0.001

LR: logistic regression; RF: random forest; SVM: support vector machine; AUC; area under curve; PPV: positive prediction value; NPV: negative prediction value; * Compared by DeLong test.

**Table 3:** Comparisons between models’ performances by Delong test.

LR	Models	**Shape Control**	**PI-RADS Alone**	**+2 Features**	**+3 Features**	**+4 Features**	**+5 Features**	**+6 Features**	**+7 Features**
	Shape Control								
	PI-RADS Alone								
	PI-RADS+2 Features	<0.001	<0.001						
	PI-RADS+3 Features	<0.001	<0.001	0.051					
	PI-RADS+4 Features	<0.001	<0.001	0.935	0.531				
	PI-RADS+5 Features	<0.001	<0.001	0.776	0.339	0.317			
	PI-RADS+6 Features	<0.001	<0.001	0.598	0.879	0.528	0.369		
	PI-RADS+7 Features	<0.001	<0.001	0.539	0.806	0.472	0.329	0.613	
RF	Models	Shape Control	PI-RADS Alone	+2 Features	+3 Features	+4 Features	+5 Features	+6 Features	+7 Features
	Shape Control								
	PI-RADS Alone								
	PI-RADS+2 Features	<0.001	0.005						
	PI-RADS+3 Features	<0.001	0.220	0.164					
	PI-RADS+4 Features	<0.001	0.042	0.688	0.280				
	PI-RADS+5 Features	<0.001	0.042	0.704	0.337	0.993			
	PI-RADS+6 Features	<0.001	0.008	0.553	0.076	0.364	0.317		
	PI-RADS+7 Features	<0.001	0.005	0.401	0.050	0.266	0.350	0.563	
SVM	Models	Shape Control	PI-RADS Alone	+2 Features	+3 Features	+4 Features	+5 Features	+6 Features	+7 Features
	Shape Control								
	PI-RADS Alone								
	PI-RADS+2 Features	0.004	<0.001						
	PI-RADS+3 Features	0.002	<0.001	0.422					
	PI-RADS+4 Features	<0.001	<0.001	0.055	0.148				
	PI-RADS+5 Features	<0.001	<0.001	0.055	0.302	0.317			
	PI-RADS+6 Features	<0.001	<0.001	0.004	0.016	0.106	0.051		
	PI-RADS+7 Features	<0.001	<0.001	0.016	0.066	0.312	0.193	0.155	

LR: logistic regression; RF: random forest; SVM: support vector machine; AUC; area under curve; PPV: positive prediction value; NPV: negative prediction value; PI-RADS: the Prostate Imaging Reporting and Data System. The performance differences between models are exhibited as the Delong test’s *p*-value. The optimal models for each algorithm are highlighted with color coding.

## Data Availability

The HQM datasets generated and analyzed in this study are available from the corresponding authors upon reasonable request. The Cancer Imaging Archive (TCIA) Database PROSTATE-US-MR Collection can be accessed online (https://www.cancerimagingarchive.net/collection/prostate-mri-us-biopsy, accessed on 23 May 2023).

## Supplementary Materials

The following supporting information can be downloaded at: https://www.mdpi.com/article/10.3390/cancers16172944/s1, Figure S1: Dataset splitted into train and validation set based on subject level. Ten-fold cross-validation least angle regression for feature selection; Figure S2: Comparison between the optimal model trained based on subject level and PI-RADS in HQM set. Column plot showing the area under curve of the logistic regression model (7 features+PI-RADS). The LR model showed a significant superior performance than PI-RADS alone (*p* < 0.001); Table S1: Train dataset and Validation dataset; Table S2: LARS Steps; Table S3: Performance of different models in TCIA dataset; Table S4: Performance of different models in HQM dataset; Table S5: Train dataset and Validation dataset based on Subject Level; Table S6: LARS Steps based on Subject Level; Table S7: Performance of different models in TCIA dataset splitted based on subject level; Table S8: Comparisons between models’ performances by Delong test based on subject level.
