# Supplementary material for: MRI T2w Radiomics-Based Machine Learning Models in Imaging Simulated Biopsy Add Diagnostic Value to PI-RADS in Predicting Prostate Cancer: A Retrospective Diagnostic Study
Source: Cancers (Basel). 2024 Aug 23;16(17):2944. doi: 10.3390/cancers16172944 (PMC11394278; doi:10.3390/cancers16172944)
Supplement: Supplementary file 1 [file cancers-16-02944-s001.zip › cancers-3150969-supplementary.pdf]

Supplementary Table S1. Train dataset and Validation dataset

	After Resample	Training	Validation	P value*
Number	718	504	214	/
Biopsy Outcom	/	/	/	0.98
Benign	355 (49.44%)	249 (49.40%)	106 (49.53%)	/
Malignant	363 (50.56%)	255 (50.60%)	108 (50.47%)	/
PI-RADS	/	/	/	0.2
Grade 1	23 (3.2%)	15 (2.98%)	8 (3.74%)	/
Grade 2	201 (27.99%)	136 (26.98%)	65 (30.37%)	/
Grade 3	135 (18.8%)	96 (19.05%)	39 (18.22%)	/
Grade 4	176 (24.51%)	122 (24.21%)	54 (25.23%)	/
Grade 5	183 (25.49%)	135 (26.79%)	48 (22.43%)	/
MRI	/	/	/	0.42
Benign	359 (50.00%)	247 (49.01%)	112 (52.34%)	/
Malignant	359 (50.00%)	257 (50.99%)	102 (47.66%)	/

PSA: prostate specific antigen; PCa: prostate cancer; GG: grade group

*Wilcoxon signed-rank test comparing training dataset and validation dataset

Supplementary Table S2. LARS Steps

	Features	MSE
Step 1	/	0.2509
Step 2	originalglcmSumEntropy	0.2248
Step 3	originalgldmDependenceNonUniformity	0.2214
Step 4	originalfirstorderMaximum	0.2183
Step 5	originalglrlmRunLengthNonUniformity	0.216
Step 6	originalglszmSizeZoneNonUniformity	0.214
Step 7	originalshapeSurfaceArea	0.2129
Step 8	originalshapeMaximum1DDiameterColumn	0.2121
Step 9	originalglcmDifferenceVariance	0.2123
Step 10	originalngtdmCoarseness	0.2128

Supplementary Table S3. Performance of different models in TCIA dataset

	Variables	AUC	Sensitivity	Specificity	PPV	NPV	p value (vs. PIRADS Control)	p value (vs. Shape Control)
LR	Shape Control	0·659 (0·596-0·723)	61.11%	70.75%	68.04%	64.10%	/	/
	PIRADS Alone	0·701 (0·640-0·763)	67.59%	72.64%	71.57%	68.75%	/	/
	2 Features	0.795 (0.779-0.89)	75.00%	66.98%	69.83%	72.45%	0.032	<0.001
	3 Features	0.801 (0.783-0.894)	75.00%	68.87%	71.05%	73.00%	0.021	<0.001
	4 Features	0.794 (0.78-0.891)	77.78%	66.98%	70.59%	74.74%	0.031	<0.001
	5 Features	0.791 (0.777-0.889)	77.78%	67.92%	71.19%	75.00%	0.036	<0.001
	6 Features	0.783 (0.784-0.896)	77.78%	66.04%	70.00%	74.47%	0.066	<0.001
	7 Features	0.784 (0.785-0.896)	77.78%	66.04%	70.00%	74.47%	0.065	<0.001
RF	Shape Control	0.617 (0.552-0.682)	58.33%	65.09%	63.00%	60.53%	/	/
	PIRADS Alone	0·701 (0·640-0·763)	67.59%	72.64%	71.57%	68.75%	/	/
	2 Features	0.650 (0.585-0.713)	70.37%	59.43%	63.87%	66.32%	0.260	0.462
	3 Features	0.645 (0.58-0.708)	70.37%	58.49%	63.33%	65.96%	0.225	0.501
	4 Features	0.687 (0.624-0.749)	73.15%	64.15%	67.52%	70.10%	0.750	0.094
	5 Features	0.687 (0.624-0.749)	72.22%	65.09%	67.83%	69.70%	0.754	0.079
	6 Features	0.729 (0.669-0.789)	74.07%	71.70%	72.73%	73.08%	0.533	0.004
	7 Features	0.701 (0.639-0.763)	69.44%	70.75%	70.75%	69.44%	0.997	0.023
SVM	Shape Control	0·655 (0·593-0·728)	55.56%	75.47%	69.77%	62.50%	/	/
	PIRADS Alone	0·701 (0·640-0·763)	67.59%	72.64%	71.57%	68.75%	/	/
	2 Features	0.715 (0.654-0.776)	72.22%	70.75%	75.21%	79.38%	0.008	0.004
	3 Features	0.687 (0.624-0.749)	69.44%	67.92%	75.21%	81.72%	0.002	0.002
	4 Features	0.696 (0.634-0.758)	74.07%	65.09%	78.26%	81.82%	<0.001	<0.001
	5 Features	0.701 (0.639-0.762)	74.07%	66.04%	78.07%	81.00%	<0.001	<0.001
	6 Features	0.729 (0.669-0.789)	74.07%	71.70%	81.42%	84.16%	<0.001	<0.001
	7 Features	0.748 (0.689-0.806)	76.85%	72.64%	81.08%	82.52%	<0.001	<0.001

LR: logistic regression; RF: random forest; SVM: support vector machine; AUC; area under curve; PPV: positive prediction value; NPV: negative prediction value

* Compared by DeLong test

Supplementary Table S4. Performance of different models in HQM dataset

	Variables	AUC	Sensitivity	Specificity	PPV	NPV	p value (vs. PIRADS Control)	p value (vs. Shape Control)
LR	Shape Control	0.577 (0.485-0.668)	26.67%	88.68%	57.14%	68.12%	/	/
	PIRADS Alone	0.658 (0.555-0.761)	46.67%	84.91%	63.64%	73.77%	/	/
	PIRADS+2 Features	0.870 (0.795-0.946)	60.00%	88.68%	75.00%	79.66%	<0.001	<0.001
	PIRADS+3 Features	0.870 (0.795-0.946)	56.67%	92.45%	80.95%	79.03%	<0.001	<0.001
	PIRADS+4 Features	0.862 (0.781-0.942)	66.67%	83.02%	68.97%	81.48%	<0.001	<0.001
	PIRADS+5 Features	0.858 (0.778-0.939)	63.33%	83.02%	67.86%	80.00%	<0.001	<0.001
	PIRADS+6 Features	0.869 (0.795-0.943)	46.67%	98.11%	93.33%	76.47%	<0.001	<0.001
	PIRADS+7 Features	0.869 (0.795-0.943)	50.00%	88.68%	71.43%	75.81%	<0.001	<0.001
	2 Features	0.852 (0.795-0.946)	73.33%	90.57%	81.48%	85.71%	0.003	<0.001
	3 Features	0.861 (0.795-0.946)	73.33%	92.45%	84.62%	85.96%	0.002	<0.001
	4 Features	0.829 (0.781-0.942)	90.00%	64.15%	58.70%	91.89%	0.006	<0.001
	5 Features	0.822 (0.778-0.939)	63.33%	86.79%	73.08%	80.70%	0.011	<0.001
	6 Features	0.864 (0.795-0.943)	83.33%	71.70%	62.50%	88.37%	<0.001	<0.001
	7 Features	0.865 (0.795-0.943)	86.67%	69.81%	61.90%	90.24%	<0.001	<0.001
RF	Shape Control	0.590 (0.479-0.701))	66.67%	54.72%	45.45%	74.36%	/	/
	PIRADS Alone	0.658 (0.555-0.761)	46.67%	84.91%	63.64%	73.77%	/	/
	PIRADS+2 Features	0.570 (0.473-0.667)	80.00%	33.96%	40.68%	75.00%	0.115	0.593
	PIRADS+3 Features	0.553 (0.453-0.653)	76.67%	33.96%	39.66%	72.00%	0.056	0.593
	PIRADS+4 Features	0.536 (0.433-0.64)	73.33%	33.96%	38.60%	69.23%	0.024	0.478
	PIRADS+5 Features	0.501 (0.397-0.605)	70.00%	30.19%	36.21%	64.00%	0.003	0.225
	PIRADS+6 Features	0.575 (0.545-0.755)	73.33%	56.60%	48.89%	78.95%	0.874	0.359
	PIRADS+7 Features	0.650 (0.606-0.789)	86.67%	52.83%	50.98%	87.50%	0.478	0.108
	2 Features	0.535 (0.460-0.610)	90.00%	16.98%	38.03%	75.00%	0.050	0.286
	3 Features	0.552 (0.483-0.620)	93.33%	16.98%	38.89%	81.82%	0.060	0.534
	4 Features	0.566 (0.520-0.612)	100.00%	13.21%	39.47%	100.00%	0.100	0.684
	5 Features	0.575 (0.527-0.624)	100.00%	15.09%	40.00%	100.00%	0.154	0.808
	6 Features	0.557 (0.514-0.600)	100.00%	11.32%	38.96%	100.00%	0.068	0.676
	7 Features	0.566 (0.520-0.612)	100.00%	13.21%	39.47%	100.00%	0.100	0.798
SVM	Shape Control	0.504 (0.412-0.595)	80.00%	20.75%	36.36%	64.71%	/	/
	PIRADS Alone	0.658 (0.555-0.761)	46.67%	84.91%	63.64%	73.77%	/	/

	PIRADS+2 Features	0.691 (0.616-0.765)	96.67%	41.51%	48.33%	95.65%	0.568	0.002
	PIRADS+3 Features	0.681 (0.607-0.756)	96.67%	39.62%	47.54%	95.45%	0.683	0.004
	PIRADS+4 Features	0.629 (0.544-0.714)	90.00%	35.85%	44.26%	86.36%	0.619	0.058
	PIRADS+5 Features	0.641 (0.55-0.732)	86.67%	41.51%	45.61%	84.62%	0.766	0.046
	PIRADS+6 Features	0.745 (0.677-0.813)	100.00%	49.06%	52.63%	100.00%	0.125	0.000
	PIRADS+7 Features	0.738 (0.663-0.813)	96.67%	50.94%	52.73%	96.43%	0.158	0.000
	2 Features	0.566 (0.520-0.612)	100.00%	13.21%	48.33%	95.65%	0.568	0.224
	3 Features	0.557 (0.514-0.600)	100.00%	11.32%	47.54%	95.45%	0.683	0.292
	4 Features	0.557 (0.514-0.600)	100.00%	11.32%	44.26%	86.36%	0.619	0.309
	5 Features	0.547 (0.507-0.587)	100.00%	9.43%	45.61%	84.62%	0.766	0.394
	6 Features	0.538 (0.502-0.574)	100.00%	7.55%	52.63%	100.00%	0.125	0.495
	7 Features	0.557 (0.514-0.600)	100.00%	11.32%	52.73%	96.43%	0.158	0.309

LR: logistic regression; RF: random forest; SVM: support vector machine; AUC; area under curve; PPV: positive prediction value; NPV: negative prediction value; PI-RADS: the Prostate Imaging Reporting and Data System

The performance differences between models are exhibited as Delong test's p-value.

Supplementary Table S5. Train dataset and Validation dataset based on Subject Level

	Training	Training after resampling	Validation	Validation after resample	P value*
Number	644	551	176	158	/
Biopsy Outcom	/	/	/	/	0.98
Benign	283 (43.94%)	268 (48.64%)	80 (45.45%)	78 (49.37%)	/
Malignant	361 (56.06%)	283 (51.36%)	96 (54.55%)	80 (50.63%)	/
PI-RADS	/	/	/	/	0.2
Grade 1	23 (3.57%)	17 (3.09%)	8 (4.55%)	7 (4.43%)	/
Grade 2	186 (28.88%)	142 (25.77%)	49 (27.84%)	41 (25.95%)	/
Grade 3	131 (20.34%)	109 (19.78%)	29 (16.48%)	26 (16.46%)	/
Grade 4	144 (22.36%)	137 (24.86%)	50 (28.41%)	47 (29.75%)	/
Grade 5	160 (24.84%)	146 (26.5%)	40 (22.73%)	37 (23.42%)	/
MRI	/	/	/	/	0.42
Benign	340 (52.8%)	268 (48.64%)	86 (48.86%)	74 (50%)	/
Malignant	304 (47.2%)	283 (51.36%)	90 (51.14%)	74 (50%)	/

PSA: prostate specific antigen; PCa: prostate cancer; GG: grade group

*Wilcoxon signed-rank test comparing training dataset and validation dataset

Supplementary Table 6. LARS Steps based on Subject Level

	Features	MSE
Step 1	/	0.251
Step 2	originalshapeMajorAxisLength	0.239
Step 3	originalshapeMinorAxisLength	0.229
Step 4	originalfirstorderKurtosis	0.226
Step 5	originalfirstorderMinimum	0.221
Step 6	originalglcmInverseVariance	0.216
Step 7	originalglcmSumAverage	0.213
Step 8	originalglcmSumSquares	0.211
Step 9	originalglrlmRunEntropy	0.211
Step 10	originalglszmLowGrayLevelZoneEmphasis	0.210
Step 11	originalglszmSmallAreaLowGrayLevelEmphasis	0.210

Supplementary Figure S1

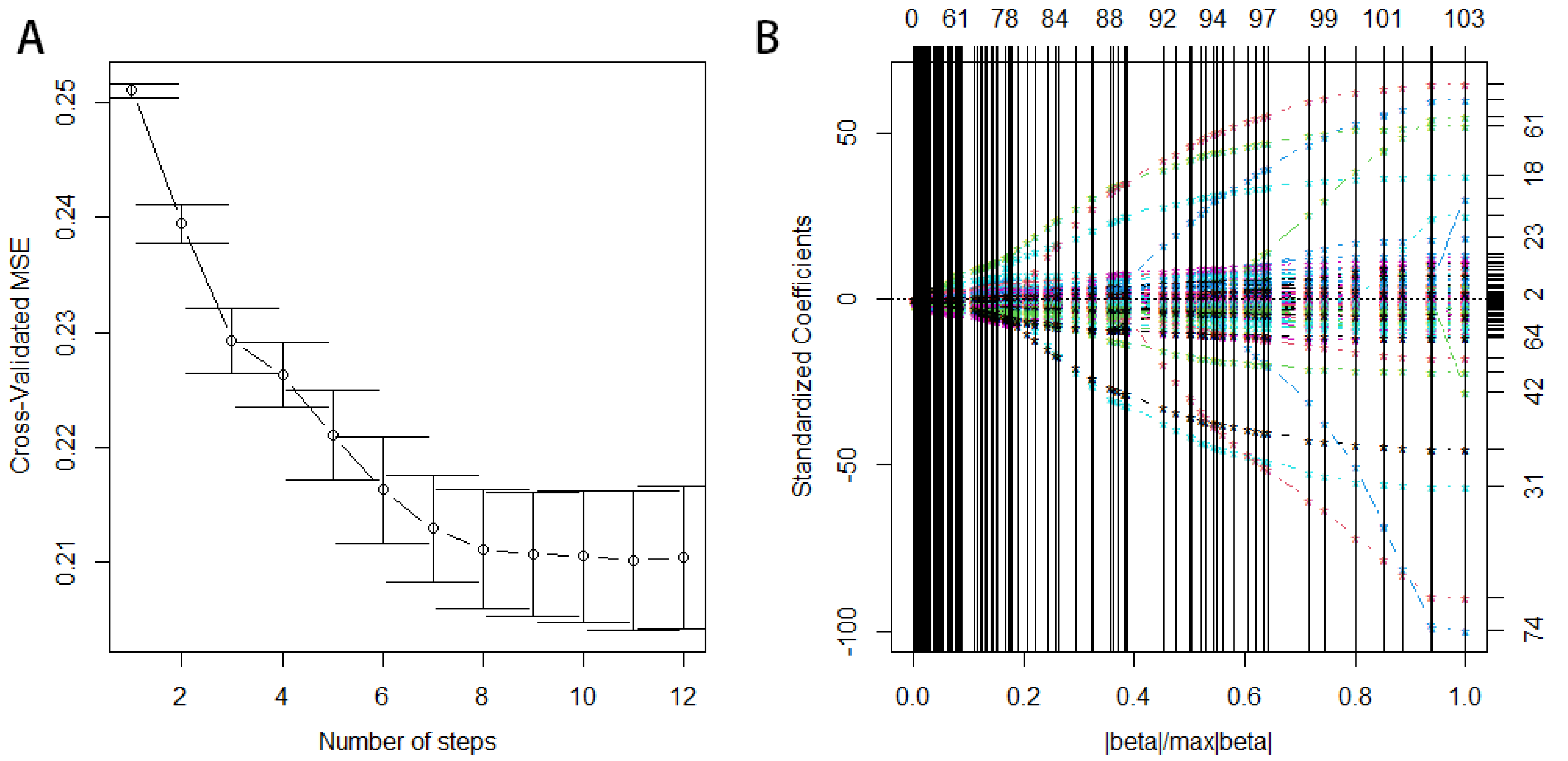

Supplementary Figure S1. Dataset splitted into train and validation set based on subject level. Ten-fold cross-validation least angle regression for feature selection. Supplementary Figure S1. Plots describing the 10-fold cross-validation least angle regression (cv-LARS) based feature selection process and exhibits the results. Figure 2A. illustrating the changes in cross-validated Mean Squared Error (MSE) with the number of steps. At the 11th step, the LARS algorithm reaches the minimum MSE. Figure 2B. exhibiting the solution path plot of 10-fold cv-LARS.

Supplementary Table S7. Performance of different models in TCIA dataset splitted based on subject level

	Variables	AUC	Sensitivity	Specificity	PPV	NPV	p value (vs. PIRADS Control)	p value (vs. Shape Control)
LR	Shape Control	0.634 (0.560-0.708)	55.00%	71.79%	66.67%	60.87%	/	/
	PI-RADS Alone	0.683 (0.610-0.756)	71.25%	65.38%	67.86%	68.92%	/	/
	PI-RADS+2 Features	0.814 (0.745-0.883)	92.50%	62.82%	71.84%	89.09%	0.001	<0·001
	PI-RADS+3 Features	0.812 (0.743-0.881)	92.50%	61.54%	71.15%	88.89%	0.002	<0·001
	PI-RADS+4 Features	0.813 (0.745-0.881)	71.25%	80.77%	79.17%	73.26%	0.001	<0·001
	PI-RADS+5 Features	0.822 (0.755-0.889)	88.75%	66.67%	73.20%	85.25%	0.001	<0·001
	PI-RADS+6 Features	0.822 (0.755-0.888)	88.75%	66.67%	73.20%	85.25%	0.001	<0·001
	PI-RADS+7 Features	0.864 (0.807-0.921)	81.25%	82.05%	82.28%	81.01%	<0·001	<0·001
	PI-RADS+8 Features	0.861 (0.803-0.918)	82.50%	78.21%	79.52%	81.33%	<0·001	<0·001
	PI-RADS+9 Features	0.860 (0.802-0.917)	82.50%	75.64%	77.65%	80.82%	<0·001	<0·001
	PI-RADS+10 Features	0.859 (0.802-0.916)	81.25%	76.92%	78.31%	80.00%	<0·001	<0·001
RF	Shape Control	0.684 (0.612-0.757)	62.50%	74.36%	71.43%	65.91%	/	/
	PI-RADS Alone	0.683 (0.610-0.756)	71.25%	65.38%	67.86%	68.92%	/	/
	PI-RADS+2 Features	0.728 (0.658-0.795)	82.50%	62.82%	69.47%	77.78%	0.164	0.430
	PI-RADS+3 Features	0.728 (0.658-0.797)	77.50%	67.95%	71.26%	74.65%	0.194	0.430
	PI-RADS+4 Features	0.728 (0.658-0.797)	76.25%	69.23%	71.76%	73.97%	0.224	0.395
	PI-RADS+5 Features	0.715 (0.644-0.784)	80.00%	62.82%	68.82%	75.38%	0.445	0.570
	PI-RADS+6 Features	0.715 (0.644-0.784)	78.75%	64.10%	69.23%	74.63%	0.432	0.499
	PI-RADS+7 Features	0.747 (0.678-0.815)	77.50%	71.79%	73.81%	75.68%	0.104	0.208
	PI-RADS+8 Features	0.759 (0.692-0.826)	80.00%	71.79%	74.42%	77.78%	0.057	0.155
	PI-RADS+9 Features	0.772 (0.706-0.837)	81.25%	73.08%	75.58%	79.17%	0.026	0.049
	PI-RADS+10 Features	0.766 (0.699-0.832)	80.00%	73.08%	75.29%	78.08%	0.046	0.091
SVM	Shape Control	0.658 (0.587-0.732)	55.00%	76.92%	70.97%	62.50%	/	/
	PI-RADS Alone	0.683 (0.610-0.756)	71.25%	65.38%	67.86%	68.92%	/	/
	PI-RADS+2 Features	0.753 (0.686-0.818)	85.00%	65.38%	71.58%	80.95%	0.010	0.085
	PI-RADS+3 Features	0.715 (0.644-0.785)	77.50%	65.38%	69.66%	73.91%	0.281	0.310
	PI-RADS+4 Features	0.766 (0.699-0.831)	82.50%	70.51%	74.16%	79.71%	0.011	0.043
	PI-RADS+5 Features	0.753 (0.686-0.818)	85.00%	65.38%	71.58%	80.95%	0.032	0.072

	PI-RADS+6 Features	0.747 (0.678-0.814)	80.00%	69.23%	72.73%	77.14%	0.086	0.091
	PI-RADS+7 Features	0.778 (0.713-0.843)	82.50%	73.08%	75.86%	80.28%	0.008	0.014
	PI-RADS+8 Features	0.797 (0.734-0.860)	83.75%	75.64%	77.91%	81.94%	0.002	0.004
	PI-RADS+9 Features	0.785 (0.72-0.848)	85.00%	71.79%	75.56%	82.35%	0.007	0.008
	PI-RADS+10 Features	0.791 (0.727-0.854)	83.75%	74.36%	77.01%	81.69%	0.004	0.005

LR: logistic regression; RF: random forest; SVM: support vector machine; AUC; area under curve; PPV: positive prediction value; NPV: negative prediction value

* Compared by DeLong test

Supplementary Table S8. Comparisons between models' performances by Delong test based on subject level

	Models	Shape Control	PI-RADS Alone	+2 Features	+3 Features	+4 Features	+5 Features	+6 Features	+7 Features	+8 Features	+9 Features	+10 Features
LR	Shape Control											
	PI-RADS Alone											
	PI-RADS+2 Features	<0·001	0.001									
	PI-RADS+3 Features	<0·001	0.002	0.423								
	PI-RADS+4 Features	<0·001	0.001	0·952	0.952							
	PI-RADS+5 Features	<0·001	0.001	0.670	0.584	0.174						
	PI-RADS+6 Features	<0·001	0.001	0.671	0.585	0.173	1					
	PI-RADS+7 Features	<0·001	<0·001	0.022	0.017	<0·001	0.004	0.004				
	PI-RADS+8 Features	<0·001	<0·001	0.039	0.032	0.001	0.008	0.007	0.515			
	PI-RADS+9 Features	<0·001	<0·001	0.041	0.033	0.001	0.009	0.009	0.486	0.766		
	PI-RADS+10 Features	<0·001	<0·001	0.049	0.039	0.002	0.012	0.011	0.536	0.785	0.899	
RF	Models	Shape Control	PI-RADS Alone	+2 Features	+3 Features	+4 Features	+5 Features	+6 Features	+7 Features	+8 Features	+9 Features	+10 Features
	Shape Control											

	PI-RADS Alone											
	PI-RADS+2 Features	0.430	0.164									
	PI-RADS+3 Features	0.430	0.194	0.981								
	PI-RADS+4 Features	0.395	0.224	0.981	0.996							
	PI-RADS+5 Features	0.570	0.445	0.737	0.744	0.669						
	PI-RADS+6 Features	0.499	0.432	0.724	0.741	0.644	0.696					
	PI-RADS+7 Features	0.208	0.104	0.582	0.61	0.414	0.225	0.287				
	PI-RADS+8 Features	0.155	0.057	0.386	0.436	0.32	0.171	0.156	0.419			
	PI-RADS+9 Features	0.049	0.026	0.227	0.25	0.163	0.045	0.068	0.103	0.420		
	PI-RADS+10 Features	0.091	0.046	0.304	0.317	0.24	0.076	0.111	0.259	0.738	0.565	
	Models	Shape Control	PI-RADS Alone	+2 Features	+3 Features	+4 Features	+5 Features	+6 Features	+7 Features	+8 Features	+9 Features	+10 Features
SVM	Shape Control											
	PI-RADS Alone											
	PI-RADS+2 Features	0.085	0.010									
	PI-RADS+3 Features	0.310	0.281	0.056								
	PI-RADS+4 Features	0.043	0.011	0.579	0.044							
	PI-RADS+5 Features	0.072	0.032	1	0.183	0.510						

	**PI-RADS+6 Features**	0.091	0.086	0.849	0.298	0.369	0.726					
	**PI-RADS+7 Features**	0.014	0.008	0.380	0.039	0.589	0.302	0.132				
	**PI-RADS+8 Features**	0.004	0.002	0.133	0.011	0.193	0.116	0.044	0.253			
	**PI-RADS+9 Features**	0.008	0.007	0.290	0.033	0.471	0.245	0.136	0.772	0.303		
	**PI-RADS+10 Features**	0.005	0.004	0.229	0.027	0.370	0.191	0.108	0.565	0.653	0.549	

Supplementary Figure S2

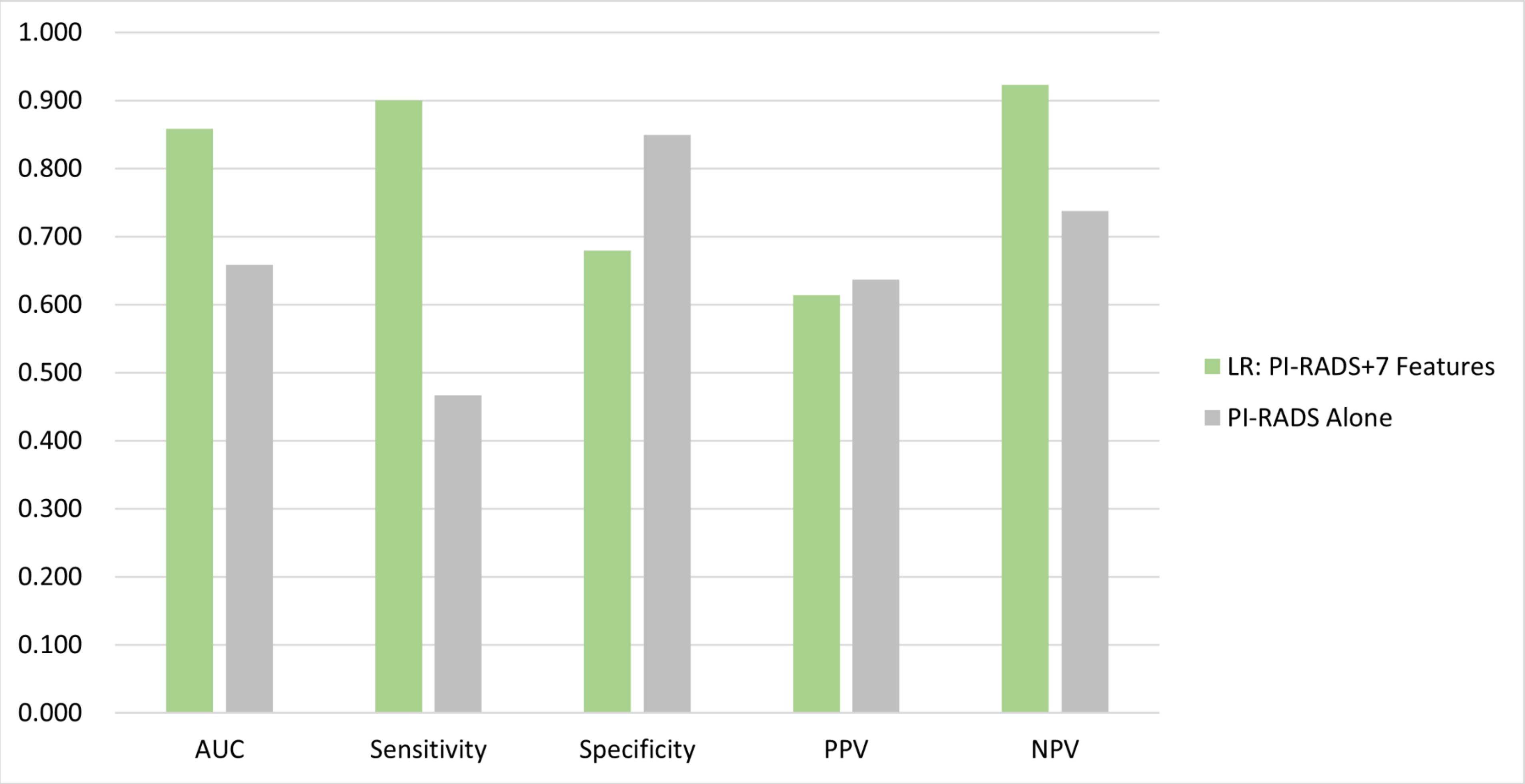

Supplementary Figure S2. Comparison between the optimal model trained based on subject level and PI-RADS in HQM set. Column plot showing the area under curve of the logistic regression model (7 features+PI-RADS). The LR model showed a significant superior performance than PI-RADS alone ($p<0.001$).
